# Data set for analyzing livestock snow disasters in the Qinghai-Tibetan Plateau

**DOI:** 10.1016/j.dib.2019.103809

**Published:** 2019-03-02

**Authors:** Tao Ye, Yijia Li, Weihang Liu, Yu Gao

**Affiliations:** a State Key Laboratory of Earth Surface Processes and Resource Ecology, Key Laboratory of Environmental Change and Natural Disaster, Ministry of Education, Faculty of Geographical Science, Beijing Normal University, Beijing 100875, China; b School of Geographic Science, East China Normal University, Shanghai 200241, China

## Abstract

This data set contains a small sample data of livestock snow disasters in the Qinghai-Tibetan Plateau, including historical loss, snow hazard measures, during disaster temperature and wind speed, and pre-disaster summer vegetation condition. This data set can be used to test/verify the method used in the method in the corresponding article published in Stoten, entitled “Linking livestock snow disaster mortality and environmental stressors in the Qinghai-Tibetan Plateau: quantification based on generalized additive models” (Y. Li et al. 2018). The data is supplied as a supplementary file attached in the research article.

**Table:** Specifications table

Subject area	*Environment*; *geography*
More specific subject area	*Disaster*
Type of data	*Links, table (.xls file)*
How data was acquired	*Livestock loss data from yearbooks;* *Meteorological data from station observations;* *Snow cover, snow depth, and vegetation index data from remoting sensing products.*
Data format	*Filtered & analyzed*
Experimental factors	*Livestock mortality of snow disaster events* *End-year livestock number (herd size)* *Duration of each snow disaster* *Max, mean and min snow cover rate during the snow disaster period* *Max, mean and min snow depth during the snow disaster period* *Max, mean, and min daily temperature during the snow disaster period* *Max daily maximum wind speed during the snow disaster period* *Annual maximum NDVI and its anomaly* *Growing season (May-Sep) aggregate precipitation and its anomaly*
Experimental features	*Data were analyzed with generalized additive models with data dredge analysis*
Data source location	*Data from various sources all adapted to county-level*
Data accessibility	*In the sample data, 30 out of the 80 records were supplied for sample analysis.* *To access to full data:* • Livestock loss data from yearbooks: partial available (1954–2008) upon request. • Meteorological data from station observations: not public repository, can contact China Meteorological Science Data Sharing Service System (CMSDS, *http://data.cma.cn/en/?r=data/detail&dataCode=A.0029.0001*) • Snow cover data: aggregated from the six-hour data provided by the National Centers for Environmental Prediction, *https://rda.ucar.edu/datasets/ds094.0/#!description*. • Snow depth data: public repository, can be found at Arid Regions Science Data Center at Lanzhou (*http://westdc.westgis.ac.cn/data/df40346a-0202-4ed2-bb07-b65dfcda9368*, in Chinese). • NDVI data: public repository, can be found at websites after registration. For GIMMS NDVI 1980–2006, please refer to the Environmental and Ecological Science Data Center for West China, National Natural Science Foundation of China *http://westdc.westgis.ac.cn/data/1cad1a63-ca8d-431a-b2b2-45d9916d860d* (in Chinese). For MODIS NDVI 2007–2015, please refer to NASA-MODIS data website for MODIS vegetation index product- Vegetation Indices 16-Day L3 Global 500 m (MOD13A1) (*https://modis.gsfc.nasa.gov/data/dataprod/mod13.php*).
Related research article	Y. Li, T. Ye, W. Liu, Y. Gao, Linking livestock snow disaster mortality and environmental stressors in the Qinghai-Tibetan Plateau: Quantification based on generalized additive models, Sci. Total Environ. 625 (2018) 87–95. https://doi.org/10.1016/j.scitotenv.2017.12.230[1]

**Table:** 

**Value of the data** • Compared to earlier studies on livestock snow disasters, great efforts have been input to make a good balance of the number of factors considered and the spatial extent of the event set. • Provided with the quality of the data, our analysis is not exhaustive. Only a generalized additive model has been applied to analyse the relationship between livestock snow disaster mortality (rate) and environment stress. Other quantitative methods can further be applied, and such a relationship can be thoroughly studied by other researchers. • This dataset can be used as a seed, and further expansion of the dataset would be extremely valuable. Livestock snow disasters occur not only in Qinghai-Tibetan Plateau, but also in the temperate steppe/meadow in the vast regions of central-to-eastern Asia. Collecting similar datasets from those regions will further allow researchers to find the commons and special issues of those regions.

## 1 Data

The data set contains the information of historical livestock snow disaster events and their corresponding meteorological conditions. It contains following data fields:

• *CODE*: event code
• *Station*: National reference meteorological station ID for the corresponding place that the disaster occurred
• *t*: The year that the disaster occurred.
• *Month1* and *day1*: the date of the snow disaster started
• *Month2* and *day2*: the date of the snow disaster ended
• *County*: Id of the county that the snow disaster occurred
• *ELE*: elevation of the county centroid. Unit: m
• *L*: loss in number (heads) in the snow disaster
• *LR*: loss rate of the snow disaster; as *L* divided by the herd size of the county at the end of the year before the disaster. Unit:%
• *N*: the herd size of the county (heads) at the end of the year before the disaster
• *NDVI*: growing season (May–Sep) maximum normalized difference vegetation index of the county before the snow disaster.
• *NDVI_a*: Anomaly of NDVI according to the time series of 1980–2012. Unit: %
• *P*: growing season (May–Sep) cumulative precipitation as recorded by the national reference meteorological station in the county before the snow disaster. Unit: mm
• *P_a*: Anomaly of *P* according to the time series of 1980–2012. Unit: %
• *Dur*: Duration of the snow disaster; as counted by the days from the starting date to the ending date of the snow disaster. Unit: d.
• *SC_min*: Minimum daily snow-cover as a percentage of land area in the county during the disaster period, calculated from satellite retrieved snow cover data (25km*25km). Unit: %
• *SC_max*: Maximum daily snow-cover as a percentage of land area in the county during the disaster period, calculated from satellite retrieved snow cover data (25km*25km). Unit: %
• *SC_mean*: Mean daily snow-cover as a percentage of land area in the county during the disaster period, calculated from satellite retrieved snow cover data (25km*25km). Unit: %
• *SD_min*: Minimum county-average daily snow-depth during the disaster period, calculated from satellite retrieved snow cover data (25km*25km). Unit: mm
• *SD_max*: Maximum county-average daily snow-depth during the disaster period, calculated from satellite retrieved snow cover data (25km*25km). Unit: mm
• *SD_mean*: Mean county-average daily snow-depth during the disaster period, calculated from satellite retrieved snow cover data (25km*25km). Unit: mm
• *T_mean*: Mean daily mean temperature during the disaster period as measured by the national reference meteorological station in the county. Unit: Celsius degree
• *T_max*: Mean daily maximum temperature during the disaster period as measured by the national reference meteorological station in the county. Unit: Celsius degree
• *T_min*: Mean daily minimum temperature during the disaster period as measured by the national reference meteorological station in the county. Unit: Celsius degree
• *V*: maximum daily mean wind speed during the disaster period as measured by the national reference meteorological station in the county. Unit: m/s

## 2 Experimental design, materials and methods

Generalized additive models (GAMs) were used to accurately predict livestock mortality and mortality rates due to snow disasters [1]. Livestock mortality (L) and mortality rates (LR) were the variables that we intended to estimate and predict. Three groups of predictors were considered: 1) snow hazard intensity, including snow disaster duration Dur, snow depth variables ($SD_{\max}$, $SD_{\min}$, and $SD_{mean}$), and snow cover variables ($SC_{\max}$, $SC_{\min}$, and $SC_{mean}$), 2) during disaster environmental stress, including wind speed v and temperature ($T_{\max}$, $T_{mean}$, and $T_{\min}$), and 3) pre-season environmental stressors concerning vegetation conditions from the previous summer, including annual maximum NDVI, NDVI, and growing season cumulative precipitation P, and their anomalies, $NDVI_{a}$ and $P_{a}$, respectively. Time trends, elevation, and herd size were considered as the controlling variables in building models. A time index variable t, which uses the year of the disaster, was considered to remove any collinearity related to time. Elevation, ELE, was considered to control for any elevation-related effects.

The fitting of GAMs includes following the steps [2]: 1) The Pearson correlation for all variables. Highly correlated predictors (with correlation coefficient >0.7) were not entered into the model simultaneously. 2) Multi-collinearity diagnostics. Variance-inflation factor (VIF) > 10 will not be considered to enter the model. 3) Finding the most promising GAMs by screening the variables of highest aggregate Akaike weights.

In our analysis, GAMs were fitted using the mgcv package of R 3.3.3 [3], [4], and the dredge analyses [5] were carried out using the MuMIn package of R 3.3.3 [6]. For each fitting, the pseudo adjusted-R^2^ and the total deviance explained were calculated as indicators of goodness-of-fit. Additionally, a 10-fold cross validation (CV) was carried out to test the prediction power of the underlying model. Metrics of predictive errors were also recorded, including the root mean square error (RMSE), the mean absolute error (MAE) and the mean error (ME).

Codes for running a GAM with dredge analysis is:

*rate52pre < -gam (log(LR)∼s(t) + s(v) + s(Dur) + s(Ele) + s(SC_mean) + s(T_mean) + s(SD_max) + s(P_a),data = d) %runing a single gam model.*

*drate52pre = dredge(rate52pre, extra = c("Rˆ2", F = function(x)summary(x)$fstatistic[[1]])) % dredge analysis, by runing all combinations of predictors, and provide the AIC results of each model as well as its adjusted R*^*2*^

*head(drate52pre, n = 20) % showing the first 20 columns of dredge results.*
